# Smooth anti-reflective three-dimensional textures for liquid phase crystallized silicon thin-film solar cells on glass

**DOI:** 10.1038/s41598-017-02874-y

**Published:** 2017-06-01

**Authors:** David Eisenhauer, Grit Köppel, Klaus Jäger, Duote Chen, Oleksandra Shargaieva, Paul Sonntag, Daniel Amkreutz, Bernd Rech, Christiane Becker

**Affiliations:** 1 Helmholtz-Zentrum Berlin für Materialien und Energie GmbH, Kekuléstr. 5, 12489 Berlin, Germany; 2 Zuse-Institut Berlin, Takustr. 7, 14195 Berlin, Germany

## Abstract

Recently, liquid phase crystallization of thin silicon films has emerged as a candidate for thin-film photovoltaics. On 10 μm thin absorbers, wafer-equivalent morphologies and open-circuit voltages were reached, leading to 13.2% record efficiency. However, short-circuit current densities are still limited, mainly due to optical losses at the glass-silicon interface. While nano-structures at this interface have been shown to efficiently reduce reflection, up to now these textures caused a deterioration of electronic silicon material quality. Therefore, optical gains were mitigated due to recombination losses. Here, the SMooth Anti-Reflective Three-dimensional (SMART) texture is introduced to overcome this trade-off. By smoothing nanoimprinted SiO_*x*_ nano-pillar arrays with spin-coated TiO_*x*_ layers, light in-coupling into laser-crystallized silicon solar cells is significantly improved as successfully demonstrated in three-dimensional simulations and in experiment. At the same time, electronic silicon material quality is equivalent to that of planar references, allowing to reach *V*_*oc*_ values above 630 mV. Furthermore, the short-circuit current density could be increased from 21.0 mA cm^−2^ for planar reference cells to 24.5 mA cm^−2^ on SMART textures, a relative increase of 18%. External quantum efficiency measurements yield an increase for wavelengths up to 700 nm compared to a state-of-the-art solar cell with 11.9% efficiency, corresponding to a j_*sc*, *EQE*_ gain of 2.8 mA cm^−2^.

## Introduction

Liquid phase crystallization (LPC) of 5–40 μm thin silicon (Si) films directly on a glass substrate is a promising technology endorsing the general trend towards reduced absorber thicknesses in silicon photovoltaics. This technique allows avoiding current challenges of silicon wafers, namely high material losses and handling issues particularly arising at very low wafer thicknesses. By scanning a line-shaped energy source, e.g. a laser beam, across silicon films on glass large-grained polycrystalline material is formed^1–4^. It has been shown that an excellent material quality equivalent to that of multi-crystalline silicon wafers can be obtained using this technology, leading to record open-circuit voltages (*V*_*oc*_) of 656 mV^5^ and 13.2% solar cell efficiency^6^. However, the short-circuit current density (*j*_*sc*_) of state-of-the-art LPC solar cells is limited by reflection losses, mainly at the planar glass-silicon interface^7^. One possibility to reduce these losses is nano- or micro-structuring of the glass-silicon interface, either by directly texturing the glass^8, 9^, or by nanoimprint lithography using high-temperature stable sol-gel films^10, 11^. While these measures efficiently enhance light in-coupling into the silicon absorber in superstrate devices, the textured interfaces cause lower material quality and higher surface recombination velocities^12^.

Similar challenges are known for other silicon thin-film technologies, e.g. nano-crystalline silicon (nc-Si) thin-film solar cells^13–16^. One successful approach for nc-Si solar cells in substrate configuration is the implementation of flat light scattering substrates (FLiSS) as light-trapping structures^17–20^. In the FLiSS approach, a periodically patterned^17^ or randomly textured^18, 19^, ZnO layer is covered by an amorphous silicon layer and subsequently polished until the tips of the ZnO pattern are bared. Using the FLiSS substrate in nc-Si solar cells lead to an equivalent material quality of the absorber compared to planar devices, and a relative increase in efficiency of 10% was observed^19^. Another technique, which was developed for amorphous silicon thin-film solar cells in superstrate configuration, uses imprinting of random nano- and micro-pyramids in a hydrogen silsesquioxane layer^21^. These pyramids are planarized by spin-coating a ZnO nanoparticle solution, thereby enabling a silicon material quality equivalent to that of planar devices while increasing the short-circuit current density, leading to an 18% relative efficiency increase.

In this contribution, we present a method for producing high-quality LPC silicon solar cells on nanostructured substrates, the SMooth Anti-Reflective Three-dimensional (SMART) texture. The SMART texture is produced by combining nanoimprint lithography of hexagonal nano-pillar arrays with spin-coating of titanium oxide layers. The spin-coating leads to a preferential filling of the voids between the hexagonal nano-pillars, resulting in a smooth surface morphology without edges and steep flanks. The refractive index contrast in the two used materials leads to an optically rough, morphologically flat texture. We present the compatibility of the SMART texture with the silicon laser crystallization and solar cell preparation process. Optical, electronic material quality and optoelectronic characterization of silicon thin-film solar cell devices on SMART textures reveal that these solar cells outperform reference cells on optimized planar interlayer systems and demonstrate how the SMART texture may improve state-of-the-art LPC silicon thin-film solar cells in combination with additional light management measures.

## Results

### Fabrication of SMART textures

**Figure 1:**
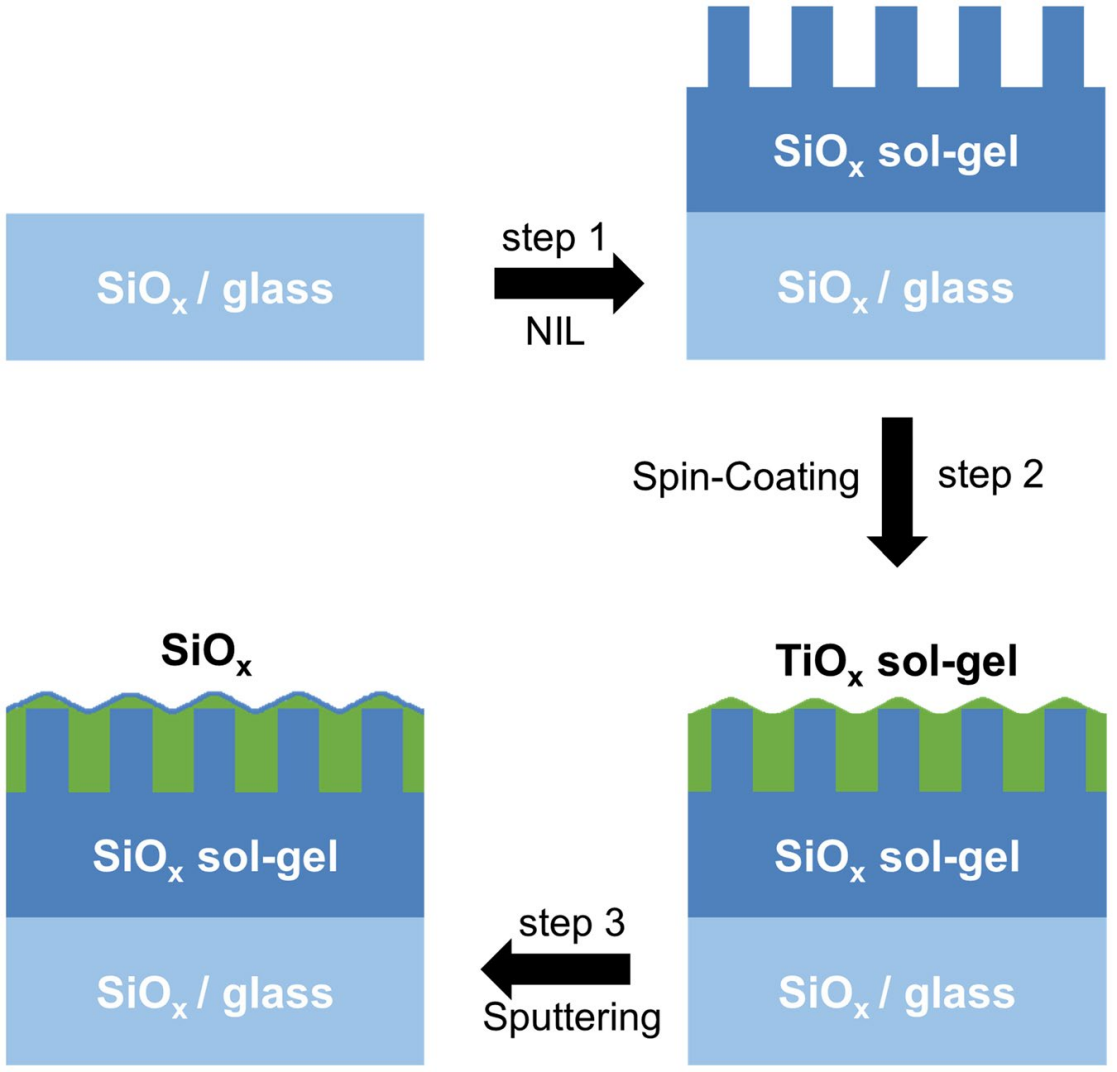
Sketch of the schematic production process of superstrates with a SMART texture.

As superstrates 1.1 mm thick Corning Eagle XG glasses with a size of 5 × 5 cm^2^ are used. 250 nm thick silicon oxide (SiO_*x*_) is sputtered onto these superstrates, serving as a diffusion barrier to glass impurities during liquid phase crystallization. Figure 1 illustrates the production process for SMART textures: Hexagonal pillar arrays with a period *p* of 750 nm and pillar heights *h* of 50 nm are replicated in a high-temperature stable, UV curable sol-gel resist based on silicon alcoxides^22^ (step 1) using nanoimprint lithography^23^. Further details about the used nanoimprint process can be found in ref. 24.

In order to smooth the surface of these superstrates, a titanium oxide precursor solution consisting of a mildly acidic solution of titanium isopropoxide in anhydrous ethanol is cast on the superstrate and spun with 2000 rpm for 30 s^25^. The spin-coating results in a preferential filling of the voids between the silicon oxide pillars, thus reducing the surface roughness significantly (step 2). By thermal curing for 30 minutes at 150 °C and 30 minutes at 500 °C, the solvents evaporate and a compact titanium oxide layer is formed^25^. Finally, a 10 nm thin silicon oxide layer is sputtered onto the stack, serving as a passivation layer at the interface with the silicon absorber (step 3).

**Figure 2:**
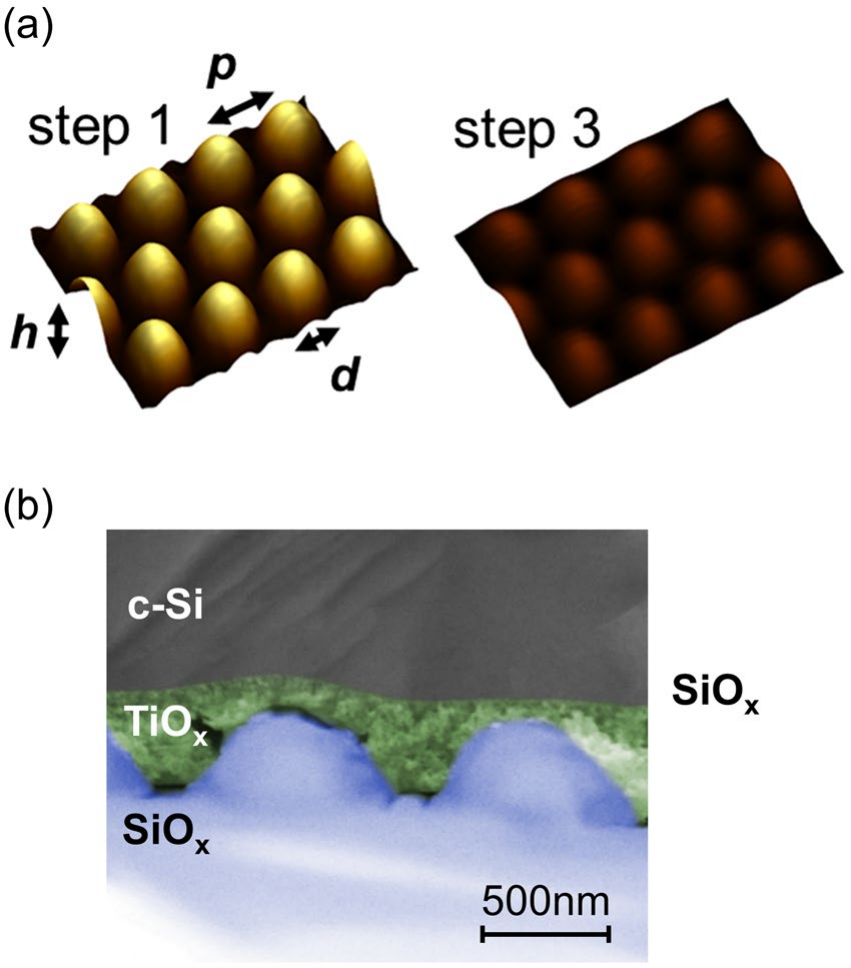
(**a**) Exemplary atomic force microscope images showing the hexagonal SiO_*x*_ nano-pillar array (cf. step 1 in Fig. 1) and the surface of the SMART texture (cf. step 3 in Fig. 1) with the same height scaling. The characteristic texture parameters are outlined. (**b**) Exemplary scanning electron microscope image of a SMART texture with a silicon absorber on top. For clarification, the SiO_*x*_ (blue) and TiO_*x*_ (green) layers have been colored. The thin SiO_*x*_ passivation layer is not visible.

Figure 2(a) shows atomic force microscope images of the hexagonal nano-pillar array (step 1) and the SMART texture after spin-coating (step 3). For comparison, the height scaling was set constant in the AFM measurements. It is clearly seen that the height of the surface protrusions from the nano-pillars is greatly reduced by spin-coating the titanium oxide. This is also observed in the scanning electron microscope image of a SMART texture cross section in Fig. 2(b). The titanium oxide (TiO_*x*_) layer (colored in green) preferably fills the voids between the SiO_*x*_ nano-pillars (colored in blue). Specifically, edges and steep flanks of the texture – which are detrimental to silicon material quality after liquid phase crystallization^11, 12^, – are flattened out to a very smooth surface morphology at the interface to the crystalline silicon (c-Si, grey).

### Simulation results

**Figure 3:**
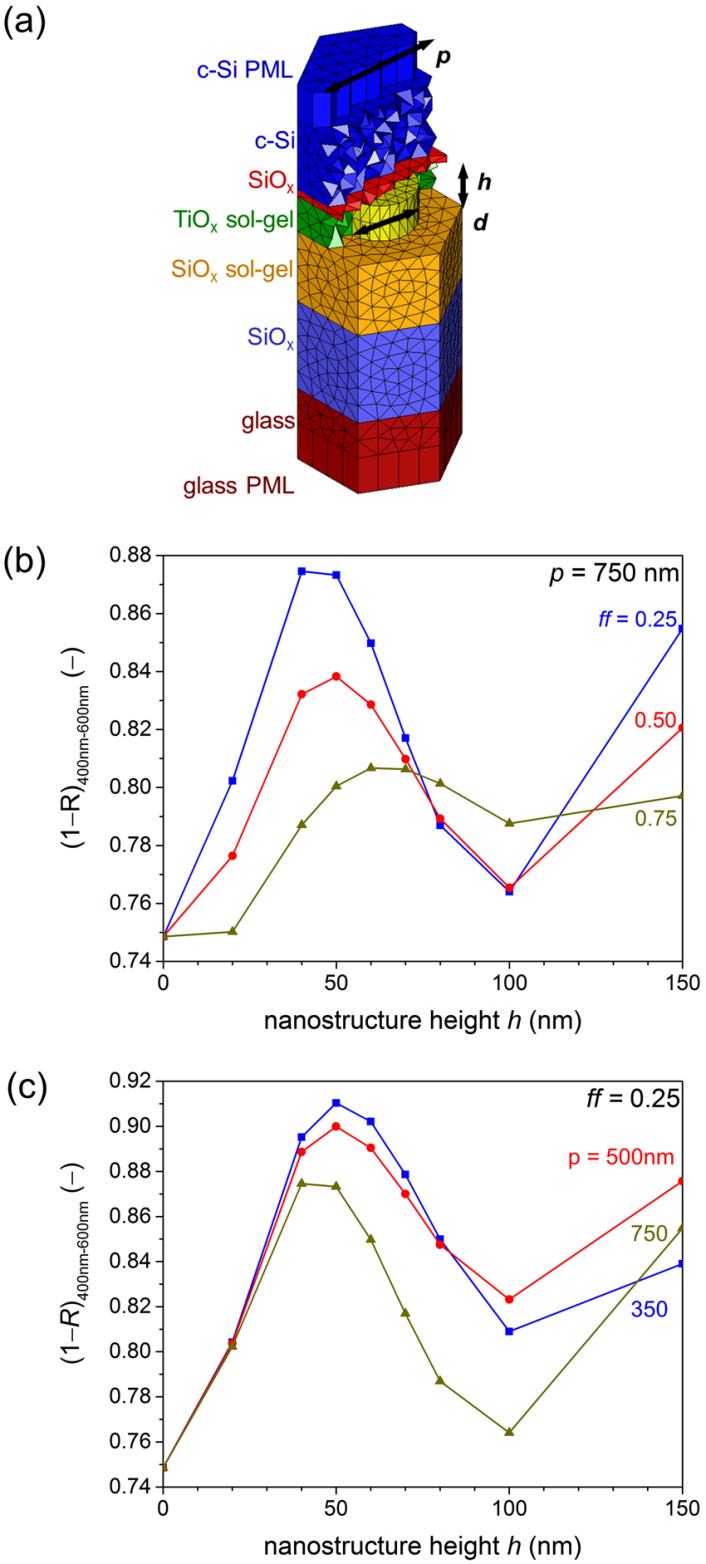
(**a**) Unit cell of the SMART texture used for 3-dimensional optical simulations, consisting of a hexagonal array of SiO_*x*_ nano-pillars and the smoothing TiO_*x*_ layer. The parameters varied in the simulations are denoted, namely the period (*p*), the height (*h*) and the diameter (**d**). On bottom and top, infinite halfspaces of glass and silicon layers are assumed, which is realized with perfectly matched layers (PML). Mean 1−*R* (reflectance) between 400 nm and 600 nm, calculated with 3-dimensional FEM simulations for (**b**) different filling fractions *ff* with a fixed period of 750 nm, and (**c**) various periods *p* of the nanostructure with a fixed filling fraction of 0.25.

In order to identify a suitable experimental structure for the SMART texture, optical simulations of hexagonal nano-pillar arrays, as sketched in Fig. 3(a), were performed with varying periods (*p*), heights (*h*) and filling fractions (*ff*). The period *p* was varied between 350 nm and 750 nm and the pillar height *h* between 20 nm and 150 nm. The filling fraction *ff* was set to 0.25, 0.5 and 0.75 by choosing the appropriate diameter *d* of the pillar following Eq. (2) (see methods section). Figure 3(b) shows the fraction of light coupled into the silicon absorber (1−*R*) in the wavelength range 400 nm–600 nm for three different filling fractions *ff* as a function of nanostructure height *h*, for a fixed period *p* of 750 nm. Smaller SiO_*x*_ nano-pillars diameters, i.e. a lower filling fraction *ff*, lead to decreased reflectance. This can be explained by interpreting the SMART texture as a single mixed medium consisting of SiO_*x*_ (*n* = 1.5) and TiO_*x*_ (*n* = 2.1). Within this representation, a smaller filling fraction of SiO_*x*_ nano-pillars corresponds to a higher effective refractive index. As the optimal refractive index between glass and silicon, given by the geometrical mean value, is around 2.4, higher effective refractive indices improve the anti-reflective properties at the interface. Filling fractions lower than 0.25 were not considered in simulations due to difficulties in the experimental production of very narrow pillars.

Therefore, *ff* was set to 0.25 during simulations of various periods (Fig. 3(c)). The simulations predict an optimal height of the SMART texture between 40 and 60 nm, which is nearly independent of the period. Comparing different periods of the nanostructure, it is seen that mean reflectance generally decreases with the period of the SMART texture. However, the reflectance difference in the optimal thickness range between the smallest (blue) and largest (gold) period is only 3%. As it has been shown that the LPC process yields better material quality for larger nanostructure periods^11^, the structure used in experiment was chosen to have a period of 750 nm, height of 45 nm and a *ff* of 0.3.

### Optoelectronic properties

**Figure 4:**
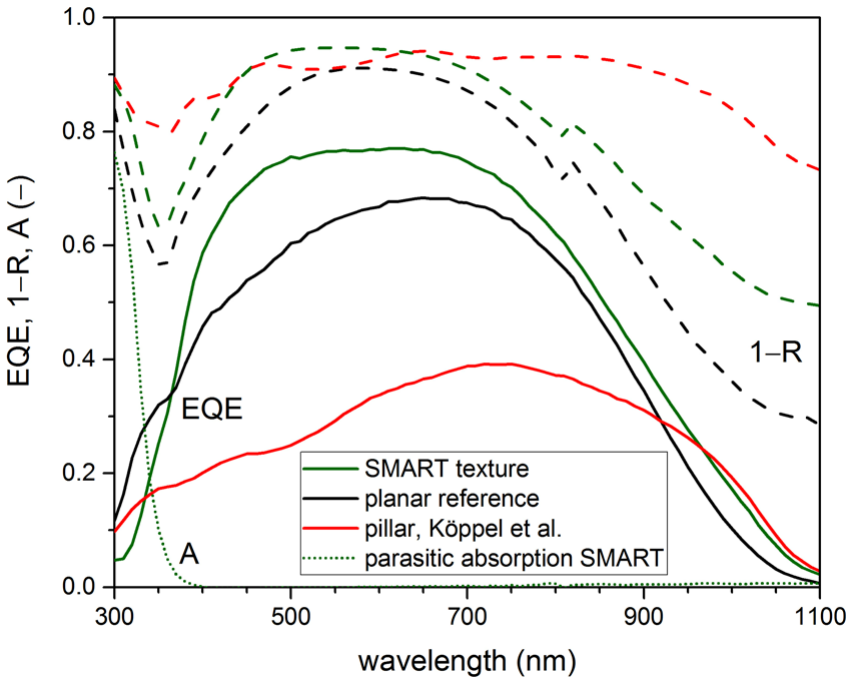
Optoelectronic characterization of solar cells with a SMART texture (green), planar reference (black) and nano-pillar array from ref. 11 (red), as measured by external quantum efficiency (EQE, solid) and 1-reflectance (1−*R*, dashed). The dotted curve shows the parasitic absorption in the SMART texture (*A*, dotted).

Optical characteristics of the prepared solar cells were measured in order to confirm their anti-reflective behavior at the glass-silicon interface. The anti-reflective properties of the SMART texture are compared to a nano-pillar array (data from ref. 11) with the same period as the SMART texture, but a higher height-to-period ratio of 0.2. The higher height-to-period ratio of the nano-pillar array is required to enable optical properties that are comparable to the SMART texture. Figure 4 shows the reflectance spectra, which are plotted as 1−*R* (dashed curves in Fig. 4). 1−*R* represents the fraction of light coupled into the absorber, where it is either absorbed or transmitted. It can be seen that both the SMART texture (green) and nano-pillar array (red) significantly reduce reflection at the glass-silicon interface in comparison to the optimized planar reference (black). These optimized planar devices exhibit an interlayer stack of 250 nm SiO_*x*_, 70 nm silicon nitride (SiN_*x*_) and 10 nm SiO_*x*_, which was previously found to have optimal broadband anti-reflective properties^2, 4^, and were processed in parallel to the SMART superstrates. Particularly, the minimum reflectance for the SMART texture is only about 5% at a wavelength of 560 nm, of which 4% (absolute) are already reflected at the sun-facing planar air-glass interface. The mean reflectance in the wavelength regime 400 nm–600 nm, where the influence of the rear side of the 8 μm thick silicon absorber can be excluded, amounts to 16% (absolute) in the planar reference stack with optimized anti-reflective SiN_*x*_ layer, and 9% (absolute) for both the nano-pillar array and the SMART texture. For longer wavelengths, the rear-side texture of the nano-pillar array leads to increased scattering at the silicon backside and higher light in-coupling.

Compared to the planar reference, light in-coupling in the long wavelength regime is enhanced for the SMART textured solar cell. As the roughness of the back silicon surface in both cases is similar as confirmed by atomic force microscope images (not shown), the increased light in-coupling is attributed to scattering of light at the SMART texture. The contrast between the refractive indices of the mixed SiO_*x*_/TiO_*x*_ layer makes it optically rough, leading to diffraction and therefore a longer light path through the absorber. Because extraction of electron-hole pairs is critical for solar cell performance, a high current density can only be reached if absorption is enhanced and at the same time, recombination remains at a low level, both in the bulk and at interfaces. Measurements of the external quantum efficiency (solid lines in Fig. 4) provide insight into carrier extraction depending on the wavelength of the incident light. It is seen that the solar cell on the nano-pillar array has a low EQE, indicating texture-induced defects at the glass-silicon interface and the bulk. This is not the case for the SMART texture (green), as its EQE is even higher compared to the parallel processed planar reference (black) for the whole wavelength range from 400 nm to 1100 nm. We attribute this to the smooth surface of the SMART texture compared to the nano-pillar array. Thus, it is confirmed that the increased light absorption in the laser-crystallized silicon absorber is not mitigated by an increased number of bulk material or interface defects. The reduced EQE in the short wavelength regime can be explained by parasitic absorption in the TiO_*x*_ layer, cf. the absorption curve (dotted) for the superstrate with a SMART texture in Fig. 4. However, solar radiation is not very strong in this wavelength regime and maximum current loss compared to a planar interlayer stack is only 0.04 mA cm^−2^. The difference between EQE and 1−*R* arises mainly due to parasitic absorption in the contact layers and recombination of carriers at interfaces and in the poly-crystalline silicon absorber. A detailed analysis of loss contributions in planar LPC silicon thin-film solar cells on glass can be found in ref. 7. In summary, the SMART texture not only enhances absorption in the 8 μm thin silicon absorber, but also preserves material quality which is vital to carrier extraction. Hence, short-circuit current density *j*_*sc, EQE*_ (cf. Eq. (1) in the methods section) could be increased from 21.0 mA cm^−2^ for the planar reference cell to 24.5 mA cm^−2^ for the solar cell with a SMART texture.

### Material quality

**Figure 5:**
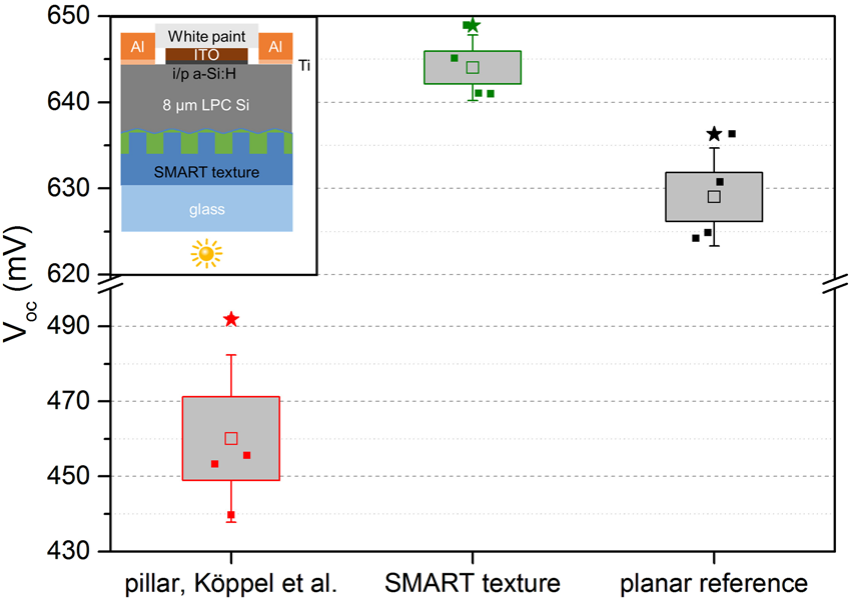
Open-circuit voltage *V*_*oc*_ of liquid phase crystallized silicon thin-film solar cell devices (see inset) obtained from Suns-*V*_*oc*_ measurements illuminated from the glass side for the planar reference sample, the SMART texture and the hexagonal nano-pillar array published in ref. 11. Open squares represent the mean value of the best four cells (solid squares), boxes the standard error, and whiskers the standard deviation. The highest measured value is indicated by a star.

Electronic material quality of the bulk LPC silicon absorbers on SMART textures and planar reference samples was evaluated by means of Suns-*V*_*oc*_ measurements. Figure 5 shows the open-circuit voltage of the four best cells (solid squares) of the planar reference, the SMART texture and the nano-pillar array from ref. 11. As for the external quantum efficiency, the bulk electronic material quality of the nano-pillar is greatly reduced. *V*_*oc*_ values for both the planar reference stack and the SMART texture samples are very high, with mean *V*_*oc*_ values of 629 mV and 636 mV for the planar reference sample and the SMART texture cells, respectively. Maximum *V*_*oc*_ values of 636 mV and 649 mV were measured. Therefore, the material quality of the bulk silicon absorber crystallized on SMART textures is at least equivalent to the planar reference stack. The increase of *V*_*oc*_ values on SMART textures cannot solely be explained by the increased current, which we estimate from a one-diode model to about Δ*V*_*oc,current*_ ≈ 3 mV. Therefore, the increased *V*_*oc*_ of silicon thin-film solar cells on SMART superstrates might be attributed to passivation properties of the TiO_*x*_ layer, which are already known from literature^26–29^. However, further investigations are needed in order to elucidate this hypothesis, e.g. using current-voltage measurements^30^.

### Solar cell characteristics

**Figure 6:**
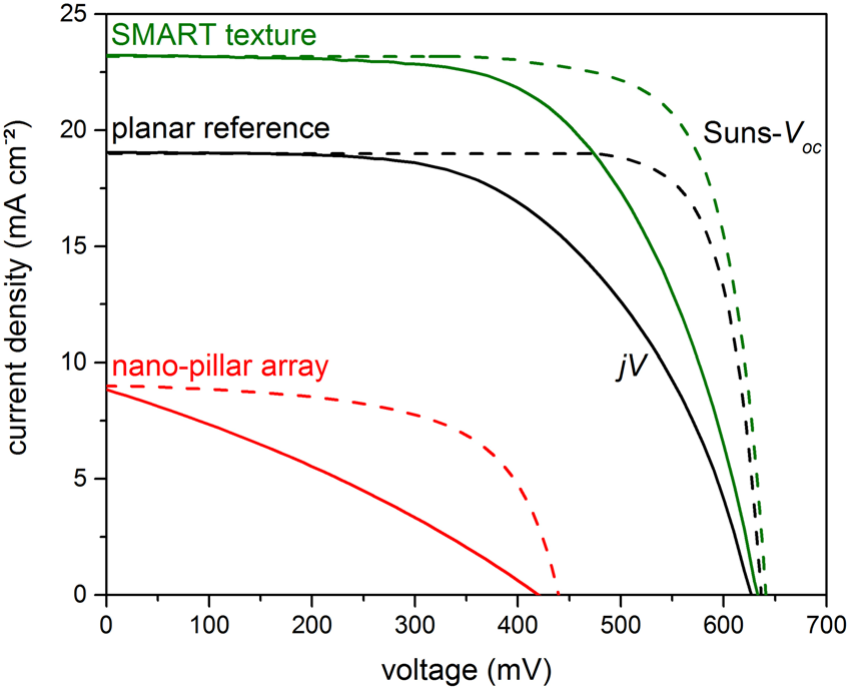
Current-voltage curves of the nano-pillar array from ref. 11 (red), the SMART texture (green) and planar reference (black), as measured with a solar simulator (solid, *jV*) and by Suns-*V*_*oc*_ (dashed).

As discussed in previous sections, implementing the SMART texture at the glass-silicon interface leads to improved optical properties of the LPC silicon solar cells while preserving material quality. Figure 6 shows the current-voltage characteristics of the best measured solar cells with a nano-pillar array (red), SMART texture (green) and planar reference (black), as measured with a solar simulator (solid lines) and calculated using Suns-*V*_*oc*_ data under the assumption of zero series resistance (dashed). While both *j*_*sc*_ and *V*_*oc*_ are strongly decreased for the nano-pillar array, the improved light in-coupling and preserved material quality of the solar cell with a SMART texture leads to a significant increase in short-circuit current density from 19.0 mA cm^−2^ in the planar reference cell to 23.2 mA cm^−2^ and equivalent open-circuit voltages of 627 mV and 633 mV, corresponding to solar cell efficiencies of 6.8% and 9.1% on the planar reference and SMART textured cells, respectively. Pronounced differences between the measured *jV* characteristics and the calculated *jV* curves from Suns-*V*_*oc*_ measurements arise from the high series resistance of the utilized solar cell processing as discussed in the methods section. Assuming zero series resistance, a pseudo power conversion efficiency of 9.7% and 11.6% can be calculated for the planar reference and SMART textures devices, respectively. Please note that neither the planar nor the SMART textured devices shown here exhibit rear side textures which would further enhance light trapping for long-wavelength light. All solar cell parameters are summarized in Table 1.

**Table 1:** Overview of solar cell parameters, maximum values obtained by *jV* characteristics of liquid phase crystallized silicon thin-film solar cells on the nano-pillar array from ref. 11, the SMART texture, parallel processed planar reference cell and back textured IBC cell from ref. 6.

Sample	*j*_*sc*_	*V*_*oc*_	*FF*	*η*	*pseudo*–*FF*	*pseudo*–*η*
	(mA cm^−2^)	(mV)	(%)	(%)	(%)	(%)
nano-pillar array^11^	8.9	420	30.4	1.1	61.1	2.4
SMART texture	23.2	633	61.8	9.1	77.7	11.6
planar reference	19.0	627	57.3	6.8	79.6	9.7
back textured IBC^6^	25.1	635	74.7	11.9		

### Comparison to state-of-the-art solar cell device

**Figure 7:**
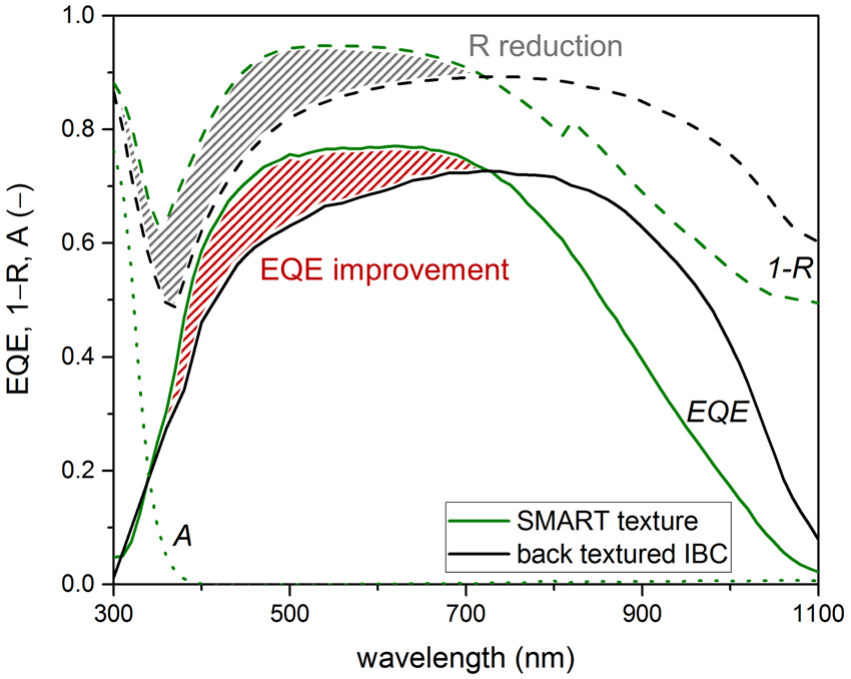
Optoelectronic characterization of the SMART texture (green) and state-of-the-art back textured IBC cell (black), measured by external quantum efficiency (EQE, solid) and 1-reflectance (1−*R*, dashed). The dotted curve shows the parasitic absorption in the SMART texture (*A*, dotted).

For further analysis of the potential of the SMART texture in high-efficiency devices, the external quantum efficiency of the best solar cell exhibiting a SMART texture is compared to a state-of-the-art LPC-Si solar cell with an interdigitated back contact (IBC) system^6^. The state-of-the-art cell exhibits a 13 μm-thick silicon absorber with n-type dopant concentration of around 1 × 10^17^ cm^−3^ and random pyramid back texture, and provides a power conversion efficiency of 11.9%^6^. If additional light-management measures at the air-glass interface are employed, a record power conversion efficiency of 13.2% was reached with this cell. In order to investigate the anti-reflective properties at the glass-silicon interface independent of other light-management techniques, the SMART texture is compared to this record cell without anti-reflective texture at the air-glass interface. Figure 7 shows the external quantum efficiency in superstrate configuration (EQE, solid lines) and reflectance (1−*R*, dashed lines) of the solar cell with a SMART texture (green) and the back textured IBC cell (black). The solar cell with a SMART texture was measured with a white-paint back reflector, while back reflection in the IBC cell is provided by the ITO/Ag contact fingers. For the IBC cell, a correction for dead areas based on a laser-beam induced current measurement (cf. supplementary material) of 8% was accounted for in order to compare the IBC cell to the full emitter cell design described in the methods section.

Comparing the reflectance (measured as 1−*R*, dashed lines) of the SMART texture (green) to the back textured IBC (black), one sees that the SMART texture efficiently reduces the reflection at the glass-silicon interface leading to increased light in-coupling up to around 700 nm (shaded in grey). For longer wavelengths, absorption in silicon is low and the optical properties are dominated by scattering at the silicon back side. Therefore, the back textured IBC cell shows improved light absorption in this wavelength regime. Considering EQE (solid lines in Fig. 7), it is seen that the improved anti-reflective properties of the SMART texture (green) at the glass-silicon interface lead to an increase in EQE compared to the IBC back textured cell (black) in the wavelength regime up to about 700 nm, corresponding to the gain in 1−*R* (shaded in red). For longer wavelengths, the EQE of the back textured IBC cell is higher due to its light-trapping texture at the silicon rear-side. From the corresponding trends in EQE and 1−*R*, it can be concluded that the internal quantum efficiency (not shown) of both, the back textured IBC cell and the solar cell on SMART texture, are equivalent, indicating excellent electronic material quality and low defect densities at the glass-silicon interface in the device exhibiting the SMART texture. Overall, *j*_*sc*,*EQE*_ amounts to 24.5 mA cm^−2^ and 26.0 mA cm^−2^ for the SMART texture and back textured IBC cell, respectively. If only contributions from the wavelength regime between 300 nm and 700 nm are considered, the current density amounts to 15.0 mA cm^−2^ and 12.2 mA cm^−2^, a 22% (relative) increase due to the SMART texture.

Solar cell parameters as obtained from current-voltage and Suns-*V*_*oc*_ measurements are summarized in Table 1. Compared to the parallel processed planar reference cell, the short-circuit current density of the solar cell on the SMART texture was increased from 19.0 mA cm^−2^ to 23.2 mA cm^−2^, corresponding to a relative increase of 18%. The open-circuit voltage is slightly higher for solar cells on SMART superstrates, which might be explained by an improved interface passivation. Both the SMART textured and planar interlayer solar cells have a *FF* of around 60% owing to the high series resistance of the contacting scheme used. From Suns-*V*_*oc*_ measurements, the *pseudo*–*FF* neglecting the influence of series resistance can be calculated, giving values close to 80%. The back textured IBC cell, in contrast, exhibits a *FF* of 74.7%. Due to its thicker absorber layer and pyramidal back texture, the short-circuit current density of the IBC cell is higher than that of the SMART textured cell (cf. Fig. 7). This leads to an overall efficiency of 11.9%.

In order to further increase the short-circuit current density and thus power conversion efficiency in liquid phase crystallized silicon thin-film solar cells, the SMART texture can be combined with additional light-management measures at the air-glass interface and silicon rear-side, as has been successfully demonstrated for planar devices^6, 7^.

For SMART textured LPC silicon absorber layers with a film thickness of 15 μm combined with a KOH random pyramid texture and a white paint back reflector at the silicon back side, analysis of the optical properties yield a maximum current density of 36.4 mA cm^−2^, indicating the potential to realize an increased short-circuit current density in LPC silicon thin-film solar cell devices^31^. Thus, an efficiency of 36.4 mA cm^−2^ × 650 mV  × 0.75 = 18% could potentially be reached for liquid-phase crystallized thin-film silicon solar cells exhibiting a SMART texture and additional light-management techniques at other interface in the device.

## Discussion and Conclusion

We presented a novel nanostructure for increased light in-coupling in liquid phase crystallized silicon thin-film solar cells: the SMooth Anti-Reflective Three-dimensional (SMART) texture. Nanoimprinted, high-temperature stable SiO_*x*_ sol-gel nano-pillars were smoothed by spin-coating of TiO_*x*_. Thereby, an optically rough nanostructure with a smooth surface could successfully be prepared and integrated in the liquid phase crystallization solar cell preparation process. Three-dimensional optical simulations allowed to obtain suitable nanostructure parameters for experiment. Anti-reflective properties and process compatibility of the nanostructure was found to be optimal at a period *p* = 750 nm, a filling fraction *ff* = 0.25 and nanostructure height *h* = 40–50 nm. Solar cells with a SMART texture were prepared using the parameters obtained from simulations. Despite its much smoother surface morphology, anti-reflective properties of the SMART texture were found to be equivalent to the more pronounced hexagonal nano-pillar array. The pronounced texture of the nano-pillar array was previously found to cause poor material quality of LPC silicon solar cells, limiting the external quantum efficiency and the open-circuit voltage due to an increased number of interface defects and extended bulk defects. In contrast, implementing the SMART texture had no detrimental effects on material quality of the silicon absorber, leading to *V*_*oc*_ values up to 649 mV and an increase in *j*_*sc*,*EQE*_ from 21.0 mA cm^−2^ to 24.5 mA cm^−2^ compared to a planar reference cell. Furthermore, the SMART texture was compared to a state-of-the-art solar cell with an interdigitated back contact system exhibiting a textured back side. Measurements of optical properties and external quantum efficiency yielded that increased light in-coupling for wavelengths up to 700 nm in the solar cell with a SMART texture was not mitigated by an increased number of defects at the glass-silicon interface. Consequently, a higher EQE on the SMART textured cell than on the state-of-the-art device that provides a power conversion efficiency of 11.9% was measured up to 700 nm, corresponding to a current gain of 2.8 mA cm^−2^ in the wavelength regime between 300 nm and 700 nm. Hence, the SMART texture allows overcoming the trade-off between optical and electronic performance in nano-structured liquid phase crystallized silicon thin-film solar cells by decoupling the anti-reflective properties from absorber structuring, potentially leading to more efficient liquid phase crystallized silicon thin-film solar cells on glass in combination with anti-reflective measures at other interfaces in future devices.

## Methods

### Solar cell preparation

The silicon absorber is deposited onto the SMART superstrates and on planar reference superstrates. The reference sample has an interlayer stack of 250 nm SiO_*x*_, 70 nm silicon nitride (SiN_*x*_) and 10 nm SiO_*x*_, which was previously found to have optimal broadband anti-reflective properties^2, 4^, and is processed in parallel to the SMART superstrates. The 8 μm thick silicon absorbers are deposited by electron beam evaporation at a heater temperature of *T* = 600 °C^32^. Liquid phase crystallization of the silicon is performed using a line-shaped laser with a 1/*e* ^2^ size of 30 × 0.3 mm^2^ and a wavelength of 808 nm. The laser beam is scanned across the samples with a scanning speed of 3 mm/s, and melts the nano-crystalline silicon layer. Upon solidifying, large grains of up to centimeters in length and millimeters in width are formed^1, 5^. Solar cells with n-type dopant concentration of about 1 × 10^17 ^cm^−3^ are prepared on both substrate types as described in refs 4 and 5, therein denoted as test structure and test cells, respectively. In this solar cell design, the LPC silicon absorber surface is cleaned using a standard RCA cleaning. Subsequently, a heterojunction is formed by depositing 5 nm intrinsic and 10 nm p-type amorphous silicon on the n-type LPC silicon absorber using low-temperature PECVD, followed by a 80 nm thick ITO layer deposited by room-temperature sputtering. Polyimide tape circles with a diameter of 8 mm are attached to the ITO, serving as etching mask in the subsequent removal of ITO and a-Si:H by wet-chemical etching. After removing native SiO_*x*_ in 1% HF solution for 30 s, 30 nm Ti and 1 μm Al is thermally evaporated on the samples. Underetching of the tape leads to separation of electron and hole contacts. Prior to measurements, the samples were annealed for 5 min at 200 °C in order to increase the mobility in the ITO layer.

Due to this quick and simple processing without lithographic techniques, these solar cell devices typically exhibit high series resistances and, consequently, have only low fill factors. Nevertheless, all vital solar cell parameters for the characterization of devices exhibiting a SMART texture at the glass-silicon interface can be measured with this contacting scheme^5^.

### Characterization

Optical characterization is conducted with a Perkin Elmer Lambda 1050 spectrophotometer equipped with an integrating sphere.

External quantum efficiency (EQE) is measured on a custom-made setup featuring a probe beam of 3 × 2 mm^2^ using lock-in technique. While no bias voltage is applied during measurements, bias light from a halogen lamp is used, imitating the AM1.5 G spectrum. Short-circuit current densities are also calculated from the EQE according to

1 $${j}_{sc,EQE}=e{\int }_{300\,nm}^{1100\,nm}EQE(\lambda )\,{\rm{\Phi }}(\lambda ){\rm{d}}\lambda ,$$

where *e* is the elementary charge and Φ(*λ*) the spectral photon flux corresponding to the AM1.5 G solar radiation spectrum.

Atomic force microscope (AFM) images were measured using a Park Systems XE-70.

Electron microscopy was performed with a Hitachi cold field emitter scanning electron microscope.

Current-Voltage characteristics are measured using a dual-source solar simulator with xenon and tungsten lamp with class AAA characteristics (WXS-155 S-L2 by Wacom Electric Co, Japan), from which open-circuit voltage and short-circuit current density can be extracted.

Alternatively, open-circuit voltages (*V*_*oc*_) are also determined using a Suns-*V*_*oc*_ unit of a WCT-100 photo conductance lifetime tool by Sinton Instruments. The setup additionally allows to calculate the so-called pseudo fill factor *pseudo*–*FF*, assuming no series resistance in the device.

### Three-dimensional optical simulations

Optical simulations are performed with the 3-dimensional finite-element method (FEM) solver JCMsuite^33^, which provides rigorous solutions to Maxwell’s equations. Sufficient numerical accuracy is achieved by constraining the side lengths of the elements to the light wavelength in the corresponding material and adapting the polynomial degree during simulations. Top and bottom of the computational domain are considered as infinite halfspaces, which is realized by using so-called perfectly matched layers (PML). By doing this, the computational cost of the simulations can be kept low^34^. On the boundaries of the computational domain periodic boundary conditions are applied. Due to the periodic structure of the SMART texture, light reflected at the glass-silicon interface can be refracted into higher orders. In order to take into account light that reaches the silicon absorber after a second light path through the glass, an *a posteriori* first order correction is applied to the reflected light as described in ref. 35. The simulated structures consist of hexagonal nano-pillar arrays, as illustrated in the meshed unit cell in Fig. 3(a). The parameters characterizing the nanostructure are the period *p*, the height *h* and the diameter *d* of the nano-pillar, as indicated in Fig. 3(a). The area filling fraction *ff* of SiO_*x*_ nano-pillars in the SMART texture is connected to *p* and *d* via

2 $$ff=\frac{\pi }{2\sqrt{\mathrm{(3)}}}{(\frac{d}{p})}^{2}$$

Light absorbed in the silicon layer or reaching the perfectly matched layer is interpreted as 1−*R* (reflectance), because the (parasitic) absorption in the interlayers can be neglected. For light that does not reach the back-side of the absorber layer due to a sufficiently short penetration depth in silicon, 1−*R* represents a measurable parameter for the characterization of the anti-reflective properties of the front interface of the device. For our devices this holds for wavelengths shorter than about 600 nm.

## Electronic supplementary material

Supplementary Information
