# Supplementary material for: Smooth anti-reflective three-dimensional textures for liquid phase crystallized silicon thin-film solar cells on glass
Source: Sci Rep. 2017 Jun 1;7:2658. doi: 10.1038/s41598-017-02874-y (PMC5453928; doi:10.1038/s41598-017-02874-y)
Supplement: Supplementary file 1 — Supplementary Information [file 41598_2017_2874_MOESM1_ESM.pdf]

Smooth anti-reflective three-dimensional textures for liquid phase crystallized silicon thin-film solar cells on glass

David Eisenhauer, Grit Köppel, Klaus Jäger, Duote Chen, Oleksandra Shargaiea, Paul Sonntag, Daniel Amkreutz, Bernd Rech, and Christiane Becker.

Supplementary material

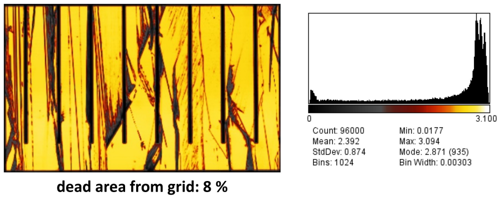

Supplementary figure 1. Laser-beam induced current measurement of the back-textured IBC solar cell.
